# Rectus Muscle Reapproximation at Cesarean Delivery and Postoperative Pain: A Randomized Controlled Trial

**DOI:** 10.1055/s-0037-1604074

**Published:** 2017-08-11

**Authors:** Deirdre J. Lyell, Mariam Naqvi, Amy Wong, Renata Urban, Brendan Carvalho

**Affiliations:** 1 Department of Obstetrics and Gynecology, Stanford University School of Medicine, Stanford, California; 2 Department of Anesthesia, Stanford University School of Medicine, Stanford, California

**Keywords:** cesarean delivery, pain, rectus closure, rectus reapproximation

## Abstract

**Objective** Rectus muscle reapproximation at cesarean delivery (CD) is performed frequently by some obstetricians; however, the effect on postoperative pain is unclear. To this end, we investigated whether rectus muscle reapproximation increases postoperative pain.

**Materials and Methods** This is a prospective, double-blind, randomized controlled trial of women undergoing primary CD with singleton or twin pregnancy at >35 weeks' gestation. Women were randomized to rectus muscle reapproximation with three interrupted sutures or no reapproximation. Exclusion criteria were prior cesarean, prior laparotomy, vertical skin incision, active labor, chronic analgesia use, allergy to opioid or nonsteroidal anti-inflammatory drugs, and body mass index ≥ 40. Intra- and postoperative pain management was standardized within the study protocol. The primary outcome was a combined movement pain and opioid use score averaged over the 72-hour study period, called the Silverman integrated assessment. Movement pain scores were assessed at 24, 48, and 72 postoperative hours.

**Results** In total, 63 women were randomized, of whom 35 underwent rectus muscle reapproximation and 28 did not. Demographic and obstetric variables were similar between groups. Silverman integrated assessment scores during the 72-hour postoperative period were higher in the rectus muscle reapproximation group (15 ± 100% vs. –31 ± 78% difference from the mean; *p* = 0.04). Operative times were similar between groups (63 ± 15 vs. 65 ± 15 minutes; *p* = 0.61), and there were no surgical complications in either group. Maternal satisfaction with analgesia at 72 hours was high in both groups (85% [73–90] rectus muscle reapproximation vs. 90% [75–100]; *p* = 0.16).

**Conclusion** Rectus muscle reapproximation increased immediate postoperative pain without differences in operative time, surgical complications, or maternal satisfaction. Benefits of rectus muscle reapproximation should be weighed against increased postoperative pain, and analgesia should be planned accordingly.

Cesarean delivery (CD) is the most common surgical procedure performed in women in the United States, with nearly 1.3 million CDs performed in 2013. 1 Rectus muscle reapproximation, or suturing of the rectus muscles, is performed by many obstetricians, including 27% of those surveyed in Canada, 2 presumably to reduce the risk of persistent rectus muscle diastasis. 3 4 However, little has been published about rectus muscle reapproximation. 5 6 When performed at a primary CD, rectus muscle reapproximation may decrease dense intra-abdominal adhesions at the subsequent repeat CD by a factor of 4. 7 Short-term effects of rectus muscle reapproximation on issues such as pain have not been reported but may be a reason for clinician reluctance to perform rectus muscle reapproximation. In a review of evidence based surgery for cesarean delivery by Berghella et al, the authors stated that “Most clinicians agree that the muscles find the right anatomic location by themselves and that suturing them together can cause unnecessary pain when the woman starts to move after surgery.” 5

Given the potential for rectus muscle reapproximation to reduce adhesions but insufficient short-term postoperative data, we assessed whether rectus muscle reapproximation at CD increases postoperative pain and analgesic use in a prospective, double-blind, randomized controlled trial.

## Materials and Methods

This study is a prospective, double-blind, randomized controlled trial conducted at Lucile Packard Children's Hospital at Stanford between September 2006 and July 2013. Women undergoing scheduled primary CD who were not in labor and consented to the trial were randomly assigned to receive rectus muscle reapproximation versus no reapproximation.

Inclusion criteria were women older than 18 years with a singleton or twin pregnancy at ≥35 weeks' gestation and with American Society of Anesthesiologists (ASA) class 1 or 2. The exclusion criteria were active labor, chronic analgesia use, vertical skin incision at cesarean, allergy to opioid or nonsteroidal anti-inflammatory drugs (NSAIDs), prior laparotomy, and class III obesity (body mass index ≥ 40). Randomization was conducted through sequentially numbered opaque envelopes generated by a study nurse in blocks through a random numbers table, in a 1:1 ratio.

Surgical techniques at CD were standardized within the study and included closure of the parietal peritoneum and two-layer uterine closure. For women randomized to rectus muscle reapproximation, surgeons were instructed to place three vertical midline interrupted sutures to reapproximate the rectus muscles. Suture type was initially standardized to 2–0 chromic catgut; however, after poor recruitment of surgeons willing to comply with this initial requirement, Monocryl (Ethicon) was also allowed. Surgeons were instructed not to discuss randomization group in the operating room so that the patient remained blinded to group assignment. All surgeries were performed by an attending physician and a resident physician.

In our institution, surgeons nearly universally perform a low-transverse skin incision using a Pfannenstiel approach, with a combination of sharp and blunt dissection to open the abdomen. The rectus muscles are dissected off the fascia, and the muscles are separated in the midline by pulling. The pyramidalis muscles are not routinely separated from the rectus sheath, unless the surgeon determines a need to create more room.

Intraoperative analgesia was standardized to spinal anesthesia using intrathecal bupivacaine 12 mg, fentanyl 10 mcg, and morphine 200 mcg. Postoperative pain management was standardized to scheduled ibuprofen 600 mg or, if the patient was unable to tolerate oral medications, ketorolac 15 mg intravenous every 6 hours, and, as needed, one to two tablets of either oxycodone 5 mg with acetaminophen 325 mg or hydrocodone 5 mg with acetaminophen 500 mg for breakthrough pain management. Postoperative pain scores were assessed per routine postpartum care by the patient's nurse; patients with pain scores ≤4/10 received one tablet of the narcotic, and patients with pain scores >4/10 received two tablets every 4 hours as needed. Patients with pain unresponsive to the aforementioned treatment could receive intravenous morphine boluses as necessary.

Verbal numeric pain scores were assessed through in-person interview in the hospital by a member of the study team at 24, 48, and 72 hours after surgery, and by telephone at 6 weeks postpartum. Women were asked to rate their pain using a 0 to 10 pain scale at rest and with movement, with 0 being no pain and 10 being the worst pain imaginable. To assess pain with movement, patients who were lying down at the time of the assessment were asked to sit up, and patients sitting at the time of the assessment were asked to stand. Maternal satisfaction with pain management was measured at 72 hours on a scale of 0 (completely unsatisfied) to 100 (completely satisfied). All members of the study team who conducted the VPS interviews were blinded to the randomization group.

Total opioid use was determined by converting administered doses of oral hydrocodone or oxycodone to “intravenous morphine milligram equivalents” using a standardized relative-potency conversion scale 8 (with 20-mg oral oxycodone or hydrocodone equivalent to 10-mg intravenous morphine) and adding total oral opioid dose to any intravenous morphine used. Total milligrams of NSAIDs and acetaminophen use were also tracked and summed.

The primary outcome was the Silverman integrated assessment pain score, an approach that allows researchers to account for both the pain score and analgesic use as a single outcome measure following surgical procedures. 9 The Silverman integrated assessment pain and opioid score is calculated by first rank ordering each patient's total opioid use (morphine milligram equivalents) and area-under-the-curve movement pain score over the 72-hour study period, then calculating a mean for both opioid use and movement pain scores, expressing both opioid use and movement pain score as percent differences from the mean, and lastly adding the percent differences from the mean for the two variables. The Silverman integrated assessment gives both variables equal weight and allows the linked interaction between opioid use and pain scores to be accounted for.

An a priori sample size calculation based on previous institutional data estimated that we required 112 patients to show a 30% difference in pain scores when rectus muscle reapproximation was performed (80% power, two-tailed, *α* of 0.05). Data were analyzed by intention to treat, and the primary outcome of combined pain scores and analgesic use was analyzed using the Silverman integrated assessment 9 as described earlier. Students' *t* -tests, Mann–Whitney U test, and Pearson's χ ^2^ were applied as appropriate to analyze demographic and secondary outcome data. Normal distribution of data was determined using visual inspection, QQ plots review, and Kolmogorov–Smirnov test. The study was approved by the Stanford University Medical Center Institutional Review Board and was registered in ClinicalTrials.gov (NCT00505362).

## Results

**Fig. 1:**
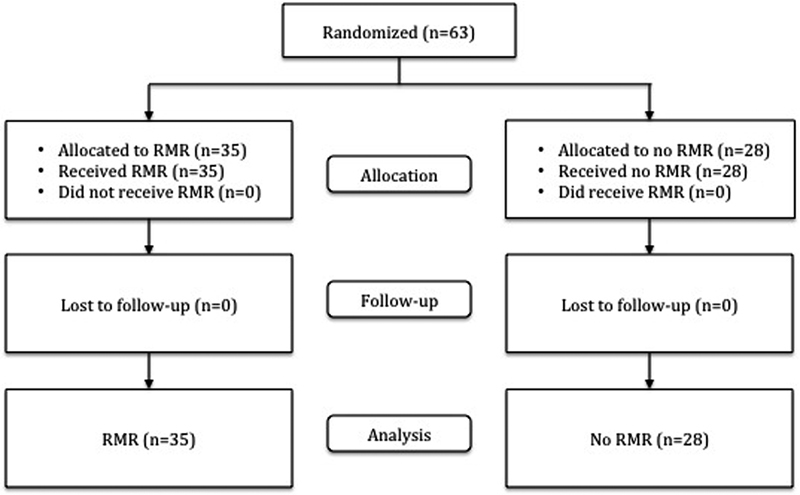
Patient enrollment flow diagram.

A total of 63 women were randomized into the study, 35 to rectus muscle reapproximation and 28 to no rectus muscle reapproximation ( Fig. 1 ). All patients received their assigned treatment, completed the study, and were analyzed in the primary outcome. The study was closed after a 6-year enrollment period, before the target sample size was obtained, because of low enrollment due to difficulties described in the discussion and to minimize potential confounding effects of a prolonged enrollment period, such as changing surgical techniques and patient population.

Demographic and obstetric variables are shown in Table 1 . Women without rectus muscle reapproximation were more likely to have had a prior full-term delivery. Other demographic and surgical variables were similar between groups.

**Table 1:** Baseline demographic and obstetric characteristics by treatment group a

	RMR ( *n* = 35)	No RMR ( *n* = 28)	*p* -Value
Age (y)	31 ± 7	33 ± 7	0.27
Race			
Caucasian	22 (63)	16 (57)	0.18
Hispanic	10 (29)	9 (32)	
Other	3(9)	3 (11)	
Parity (predelivery)			
Full term	1 (0–1)	1 (1–2)	0.01
Preterm	0 (0–0)	0 (0–0)	0.51
TAB or SAB	1 (0–1)	0 (0–1)	0.25
Living children	1 (1–1)	1 (1–2)	0.07
Gestational age (wk)	39 (37–39)	38 (37–39)	0.33
Use of staples	14 (40)	13 (46)	0.62
Twins	5 (14)	2 (7)	0.49
Cesarean Indication			
Breech	18 (51)	13 (46)	0.04
Fetal abnormality b	4 (11)	9 (32)	
Placental abnormalities c	1 (3)	7(25)	
Other d	12(34)	1(4)	

Abbreviations: RMR, rectus muscle reapproximation; SAB, spontaneous abortion; TAB, termination abortion.

a Data are presented as mean ± standard deviation, median (interquartile range), and *n* (%), where indicated, based on two-tailed Students' *t* -test, Mann–Whitney U test, and Pearson's χ ^2^ .

b Includes oligohydramnios and fetal heart rate abnormalities.

c Includes previa, low-lying placenta, and velamentous cord insertion.

d Includes preeclampsia, uterine anomalies, cesarean on maternal request, HIV, and so on.

Silverman integrated assessment scores during the 72-hour postoperative period were higher in the rectus muscle reapproximation group (15 ± 100% vs. –31 ± 78%; *p* = 0.04; Table 2 ). When assessed independently, total opioid use was similar between groups (30 mg [18–45] rectus muscle reapproximation vs. 20 mg [12–35]; *p* = 0.15; Table 2 ) Pain scores at rest and movement are shown in Table 2 . No differences in rest and movement pain scores during any time-point at 24, 48, and 72 hours were found. Women interviewed at 6 weeks postpartum reported similarly infrequent rates of incisional pain, pain at rest, and pain with movement ( Table 2 ). Our posthoc power analysis revealed 80% power to detect a 30% difference in Silverman integrated assessment scores ( *p* = 0.05).

**Table 2:** Outcomes by treatment group in the 72-hour study period a

	RMR ( *n* = 35)	No RMR ( *n* = 28)	*p* -Value
SIA score	15 ± 100	−31 ± 78	0.04
Total opioid use (mg) b	30 (18–45)	20 (12–35)	0.15
Patients requiring IV opioids	6 (17)	3 (11)	0.10
Total NSAIDs (g)	6.6 (4.8–7.8)	6 (4–7.8)	0.42
Total acetaminophen (g)	7.1 ± 4.1	6.2 ± 4.1	0.80
Rest NVPS (AUC: 0–72 h)	120 (72–192)	120 (48–189)	0.71
Movement NVPS (AUC: 0–72 h)	288 (192–384)	216 (147–330)	0.09
Any incisional pain (6 wk)	7 (20)	3 (11)	0.20
Rest VNPS (6 wk)	0 (0–0)	0 (0–0)	0.57
Movement VNPS (6 wk)	0 (0–1)	0 (0–0)	0.52

Abbreviations: AUC, area under the pain intensity x time curve; IV, intravenous; NSAIDs, nonsteroidal anti-inflammatory drugs; RMR, rectus muscle reapproximation; SIA, Silverman integrated assessment; VNPS, verbal numeric pain score (0–10, with 0 = no pain and 10 = worse pain imaginable).

a Data are presented as mean ± standard deviation, median (interquartile range), and *n* (%), where indicated, based on two-tailed Students' *t* -test and Mann–Whitney U test.

b Oral hydrocodone and oxycodone were converted to IV morphine milligram equivalents; conversion ratio: 20 mg PO oxycodone, or hydrocodone 10 mg IV morphine added to IV morphine for total.

Note: SIA of combined opioid use and movement pain score over the 72-hour study period, percentage difference from the mean ± standard deviation.

Operative times and surgical and infectious complications were similar between groups ( Table 3 ). There were no cases of wound dehiscence, cellulitis, hematoma, seroma, abscess, organ damage, or infectious morbidity in either group, and pre- and postoperative hematocrits were similar between groups. Maternal satisfaction with analgesia was high in both groups (85% [73–90] rectus muscle reapproximation vs. 90% [75–100]; *p* = 0.16).

**Table 3:** Operative and other variables a

	RMR ( *n* = 35)	No RMR ( *n* = 28)	*p* -Value
Operative time (min)	63 ± 15	65 ± 15	0.61
Surgical or infectious morbidity	0	0	
Satisfaction (0–100 scale)	85 (73–90)	90 (75–100)	0.16
Preoperative hematocrit	36.7 ± 2.1	36.1 ± 3.1	0.38
Postoperative hematocrit	31 ± 3.3	29.8 ± 4.1	0.21

Abbreviation: RMR, rectus muscle reapproximation.

a Data are presented as mean ± standard deviation and median (interquartile range), where indicated, based on two-tailed Students' *t* -test and Mann–Whitney U test.

## Comment

Our study suggests that rectus muscle reapproximation at primary CD results in increased Silverman integrated assessment scores during the first 72 postoperative hours. Increased postoperative pain with movement has been suggested as a reason some obstetricians avoid rectus muscle reapproximation, 5 and our findings appear to provide evidence for this assumption.

The evidence base to assess rectus muscle reapproximation is quite limited. In 2012, Encarnacion and Zlatnik concluded in a review of evidence-based CD that additional research is needed to assess rectus muscle reapproximation as no studies on the practice could be identified. 10 In 2013, Dahlke et al concluded in an updated systematic review of randomized controlled trials assessing techniques at CD that evidence is insufficient to assess the balance of benefits and harms for the practice of rectus muscle reapproximation. 6 A prospective cohort study of surgical techniques and adhesions, published in 2012, found a potential benefit of rectus muscle reapproximation with a reduction in dense and filmy adhesions at repeat CD. 7 To our knowledge, this study is the first to examine the relationship between rectus muscle reapproximation and pain; a PubMed search of the literature with the terms *rectus* , *cesarean* , and *pain* from 1960 to 2016 did not identify any related studies.

The frequency with which rectus muscle reapproximation is performed at CD in the United States is unknown. Rectus muscle reapproximation was performed among 24% of CDs at a single U.S. hospital in the previously mentioned study of rectus muscle reapproximation and adhesions. 7 In a Canadian survey, 27% of respondents reported routinely performing rectus muscle reapproximation. 2 Given the nearly 1.3 million CDs performed in the United States in 2013, 1 it is likely that rectus muscle reapproximation is practiced in a large number of women even if one assumes the percentage of obstetricians employing the practice is relatively low.

Our study highlights the importance of using a tool such as the validated but not widely used Silverman integrated assessment to assess pain and opioid use, as it accounts for critical interactions between two commonly used endpoints in surgical pain studies, the pain score and opioid use, which could skew study outcomes. 9 11 For example, patients may use more opioids because they are experiencing more pain (high opioid use, high pain scores), or have lower pain scores because of additional opioid use (high opioid use, low pain scores), or have more pain due to inadequate opioid use (low opioid use and high pain scores).

Our study protocol standardized factors that could affect postoperative pain. Study participants received consistent intraoperative anesthesia, standardized postoperative pain management, and similar surgical techniques to reduce the number of potentially confounding variables and to isolate the effect of rectus muscle reapproximation on pain. As most surgeons in our institution use a Pfannenstiel skin incision and frequently a combination of blunt and sharp dissection to open the abdomen, it would have been impossible to quantify the degree to which each was used, which is why we chose a randomized study design. Closure of the parietal peritoneum has been shown to increase immediate postoperative pain 12 13 14 15 and reduce adhesion formation, 16 and as such, the parietal peritoneum was closed in all patients. Chromic catgut was initially chosen for rectus muscle reapproximation due to its relatively rapid reabsorption and absence of data suggesting increased pain at laparotomy. Closure of the skin with suture or staples was left to the surgeon's discretion, and this has been shown in meta-analysis not to alter pain. 17

We excluded patients with factors that may independently influence postoperative pain or surgical complications, including labor, chronic analgesia use, vertical skin incision at cesarean, prior laparotomy, and class III obesity. We made some assumptions where literature is absent about which factors alter pain: we included women carrying a singleton or twin gestation and excluded women delivering prior to 35 weeks' gestation in case potential emotional strains of an unexpected preterm birth influence perception of pain.

Women who underwent rectus muscle reapproximation were more likely to have a placental abnormality as the indication for CD, and were less likely to be parous or have breech presentation. Whether these differences in obstetric characteristics mattered to the primary outcome is unknown.

We encountered several challenges in running this study. Enrollment was slow. Our limited inclusion of only women undergoing a primary CD, not in labor, without prior laparotomy and without class III obesity contributed to slow enrollment. Furthermore, we standardized surgical techniques to reduce the number of potentially confounding variables and failed to anticipate that due to this some surgeons preferred not to enroll their patients. In an attempt to increase acceptance of our study by surgeons, we removed the requirement for use of 2–0 chromic catgut for rectus muscle reapproximation that some obstetricians found objectionable. There are no data suggesting that suture type alters pain when used in the abdomen, and because the study was randomized, we do not feel that this change introduced bias. Our group sizes were different despite 1:1 randomization. Early in the study, our randomization box, which was kept in a shared room and contained sequentially numbered cards, was moved and several cards were lost. We monitored that all remaining cards were opened in the correct order following this event, and verified that all surgeons complied with randomization throughout the study. However, our final group sizes were consequentially imbalanced at 28 and 35 patients, respectively. Finally, some patients were reluctant to enroll in an interventional trial where the outcome involved an assessment of pain.

Due to slow enrollment, we closed our study prematurely, after 6 years and before the target sample size was obtained, to minimize potential confounding effects inherent to a prolonged enrollment period such as changing surgical practices, suture preferences and patient population.

Nonetheless, we felt the results of this study are important to present. Rectus muscle reapproximation is not uncommon, yet data regarding postoperative pain and surgical outcomes are limited. Despite the premature termination of our study and our relatively small final patient numbers, we found a difference in combined pain and opioid use, and our post hoc power calculation revealed that we had 80% power to identify a 30% difference in Silverman integrated assessment scores between groups likely due to a better than expected effect size in the study. Our a priori power calculation was based on institutionally derived data estimates prior to starting the study due to the lack of literature on which to base it, and appears to have underestimated the effect size and therefore overestimated of the numbers needed to conduct this study.

Despite our study limitations, we feel that our rigorous methodology, with careful standardization of intra- and postoperative pain management and standardization of surgical techniques, enhanced our ability to isolate and assess rectus muscle reapproximation and its impact on postoperative pain and analgesic use. We used a standardized assessment of pain scores based on in-person pain assessment that considered movement as well as rest pain at standardized postoperative time points. Standardized analgesic pain management protocols also allowed for accurate assessments of postoperative opioid use.

Our results suggest that rectus muscle reapproximation modestly increases short-term postoperative opioid use and movement pain among women undergoing primary CD. Operative time, surgical complications, and maternal satisfaction do not appear to be impacted by rectus muscle reapproximation. The benefits described with rectus muscle reapproximation such as less adhesions, should be weighed against the potential for a modest increased postoperative pain and corresponding opioid use.
